# Profiling the cell walls of seagrasses from A (*Amphibolis*) to Z (*Zostera*)

**DOI:** 10.1186/s12870-022-03447-6

**Published:** 2022-02-04

**Authors:** Lukas Pfeifer, Gijs van Erven, Elizabeth A. Sinclair, Carlos M. Duarte, Mirjam A. Kabel, Birgit Classen

**Affiliations:** 1 Pharmaceutical Institute, Department of Pharmaceutical Biology, Christian-Albrechts-University of Kiel, Gutenbergstr. 76, 24118 Kiel, Germany; 2 Laboratory of Food Chemistry, Wageningen University & Research, Bornse Weilanden 9, 6708 WG Wageningen, The Netherlands; 3 School of Biological Sciences and Oceans Institute, University of Western Australia, Crawley, WA Australia; 4 Red Sea Research Center (RSRC) and Computational Bioscience Research Center (CBRC), King Abdullah University of Science and Technology, Thuwal, Saudi Arabia

**Keywords:** Seagrass, Cell wall, Polysaccharide, Lignin, Apiogalacturonan, Pyrolysis, Gas chromatography

## Abstract

**Background:**

The polyphyletic group of seagrasses shows an evolutionary history from early monocotyledonous land plants to the marine environment. Seagrasses form important coastal ecosystems worldwide and large amounts of seagrass detritus washed on beaches might also be valuable bioeconomical resources. Despite this importance and potential, little is known about adaptation of these angiosperms to the marine environment and their cell walls.

**Results:**

We investigated polysaccharide composition of nine seagrass species from the Mediterranean, Red Sea and eastern Indian Ocean. Sequential extraction revealed a similar seagrass cell wall polysaccharide composition to terrestrial angiosperms: arabinogalactans, pectins and different hemicelluloses, especially xylans and/or xyloglucans. However, the pectic fractions were characterized by the monosaccharide apiose, suggesting unusual apiogalacturonans are a common feature of seagrass cell walls. Detailed analyses of four representative species identified differences between organs and species in their constituent monosaccharide composition and lignin content and structure. Rhizomes were richer in glucosyl units compared to leaves and roots. *Enhalus* had high apiosyl and arabinosyl abundance, while two Australian species of *Amphibolis* and *Posidonia*, were characterized by high amounts of xylosyl residues. Interestingly, the latter two species contained appreciable amounts of lignin, especially in roots and rhizomes whereas *Zostera* and *Enhalus* were lignin-free. Lignin structure in *Amphibolis* was characterized by a higher syringyl content compared to that of *Posidonia*.

**Conclusions:**

Our investigations give a first comprehensive overview on cell wall composition across seagrass families, which will help understanding adaptation to a marine environment in the evolutionary context and evaluating the potential of seagrass in biorefinery incentives.

**Supplementary Information:**

The online version contains supplementary material available at 10.1186/s12870-022-03447-6.

## Background

Seagrasses are an evolutionary unique, polyphyletic group of angiosperm plants, which evolved early in the evolution of monocotyledonous plants, and colonized the sea more than 100 million years ago [1]. This adaptation to the marine environment was performed in three to four independent lineages [2], which gave rise to the four major seagrass families. Strikingly, despite their independent evolutionary routes, seagrasses from the different lineages have evolved many morphological similarities, which indicates that the marine surrounding imposes selection forces that can lead to convergent evolution [3]. All seagrasses belong to the order Alismatales and comprise around 70 species within these families (Posidoniaceae, Zosteraceae, Hydrocharitaceae, and Cymodoceaceae). The Posidoniaceae are monogeneric, the Zosteraceae consist of four genera (*Heterozostera, Phyllospadix, Nanozostera and Zostera*), the Hydrocharitaceae include some freshwater and three marine (*Enhalus, Halophila*, and *Thalassia*) genera and the Cymodoceaceae exhibit the highest variety of genera (*Amphibolis*, *Cymodocea*, *Halodule*, *Syringodium* and *Thalassodendron*) [1]. The genus *Ruppia*, which occurs in brackish water, is sometimes classified in the Cymodoceaceae [4], but the Angiosperm Phylogeny Group IV System [5] uses the traditional assignment to Ruppiaceae. Species of *Ruppia* are not restricted to marine waters and show a high variability in accepted salinity gradients [6–8]. They are not always accepted as seagrasses *sensu stricto*, but are included in our research because of their ecological similarity to seagrasses.

Seagrasses form important, highly productive coastal ecosystems worldwide (except Antarctica) and aid the stabilisation of sediments. Together with macroalgae, seagrasses are the dominant primary producers in coastal waters. They provide structural habitat and feeding grounds for diverse and abundant biological communities, including threatened organisms like dugongs, turtles and sea horses [9–11]. Despite their vital role, human pressures have led to significant declines of seagrass meadows, prompting the need for protection and restoration. Furthermore, large amounts of seagrass litter is washed onto beaches. Seagrasses could, therefore, form an interesting alternative feedstock for utilization in a biobased economy, not competing for arable land, that could support production of biofuels and chemicals [12].

The existing literature on adaptation of these angiosperms to the marine environment is surprisingly limited, despite the ecological importance of seagrass species. Genome sequencing of two *Zostera* species suggested that adaptation to the marine habitat was accompanied by a reduction in gene numbers of some genes involved in cell wall recycling and modification in comparison to *Arabidopsis* and *Oryza* [13, 14], though actual cell wall compositional analysis was not performed. In fact, very little is known about the cell wall composition and architecture of seagrasses (for review see [15]). Cellulose (e.g. [16]) and hemicelluloses (e.g. [17]) are considered the main components of cell walls in terrestrial and marine plants, although detailed studies on characterization of distinct (hemicellulosic) polysaccharide structures/populations are missing. Unique structural features were identified in some seagrass species and include sulfated polysaccharides rich in galactosyl (Gal) units [18–20] and unusual pectic polysaccharides mainly composed of galacturonyl (GalA) and apiosyl (Api) units (apiogalacturonans, [21]).

The secondary walls of vascular terrestrial plants also contain the cross-linked aromatic polymer lignin. Lignin is formed by radical coupling of the *p*-hydroxycinnamyl alcohols *p*-coumaryl alcohol, coniferyl alcohol and sinapyl alcohol which, respectively, give rise to *p*-hydroxyphenyl (H), guaiacyl (G) and syringyl (S) units, when incorporated into the lignin polymer [22]. The composition and structure of the lignin population depends largely on the botanical origin, but also varies substantially within plant species, e.g. as an effect of age or tissue/organ type [22, 23].

Lignin covalently and non-covalently interacts with the cell wall polysaccharides and thereby plays a pivotal role in mechanically strengthening of plant cell walls [24]. However, the question of whether seagrass cell walls contain lignin is still under debate [25–28]. One of the reasons for this discussion is that methodologies to quantify and qualify lignin polymers have been based on non-specific and non-sensitive gravimetric analysis [29, 30]. The presence of lignin was detected in some seagrass species by more detailed analysis using pyrolysis gas chromatography mass spectrometry (pyrolysis-GC–MS) [29, 31–33]. This technique allows for specific lignin quantification and structural characterization by employing uniformly ^13^C labelled lignin internal standards (^13^C-IS), opening up new possibilities to map the presence of seagrass lignin in more detail [34, 35]. Detailed NMR analyses of cell walls in *Posidonia australis* and *Posidonia oceanica* showed that sheath tissue of the latter species contained a unique, heavily *p*-hydroxybenzoylated lignin structure [36, 37]. This feature might relate to the seagrasses’ habitat, and could provide an untapped resource of novel biobased aromatics.

**Fig. 1:**
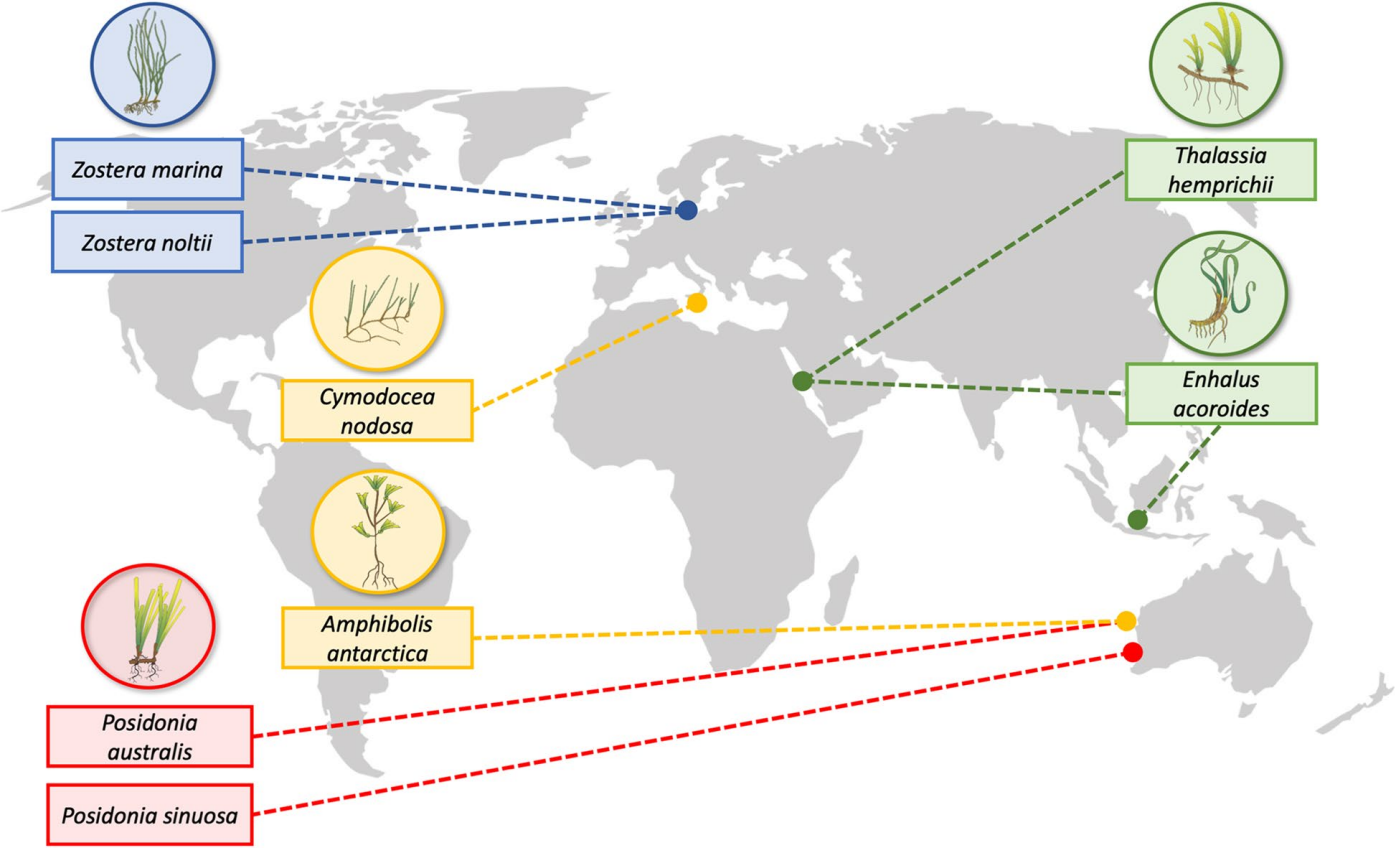
Sampling location of seagrass species investigated in this study. Families: blue: Zosteraceae; green: Hydrocharitaceae; yellow: Cymodoceaceae; red: Posidoniaceae. Plant vector symbols were used from Catherine Collier (James Cook University) and Tracey Saxby (Integration and Application Network) from the webpage https://ian.umces.edu/media-library under the license CC BY-SA 4.0 and integrated in this figure (distributed under the same license; https://creativecommons.org/licenses/by-sa/4.0/)

Incorporation of lignin into the cell wall as a common feature among seagrass families, however, is not yet confirmed. Likewise, the question whether some commonality exists in the cell wall polysaccharide composition, architecture and organisation among seagrass families remains unanswered. Here, we investigated the cell walls of seagrasses by examination of the polysaccharide composition and lignin content and structure across different families and locations. We analyzed nine species sampled from the Baltic Sea (*Ruppia* spp., Ruppiaceae; *Zostera marina* and *Zostera noltii*, Zosteraceae), the Mediterranean Sea (*Cymodocea nodosa*, Cymodoceaceae), the Red Sea (*Enhalus acoroides* and *Thalassia hemprichii*, Hydrocharitaceae) and the Indian Ocean (*P. australis* and *Posidonia sinuosa*, Posidoniaceae and *Amphibolis antarctica*, Cymodoceaceae) (Fig. 1, detailed information in Table S1). The studied species cover the four seagrass families, including the brackish water family of Ruppiaceae, as well as the three groups of seagrasses which are regarded to have evolved independently [2].

## Results

### Sequential extraction of polysaccharide fractions from nine seagrass species

#### Comparison of the constituent carbohydrate composition of the different fractions

**Fig. 2:**
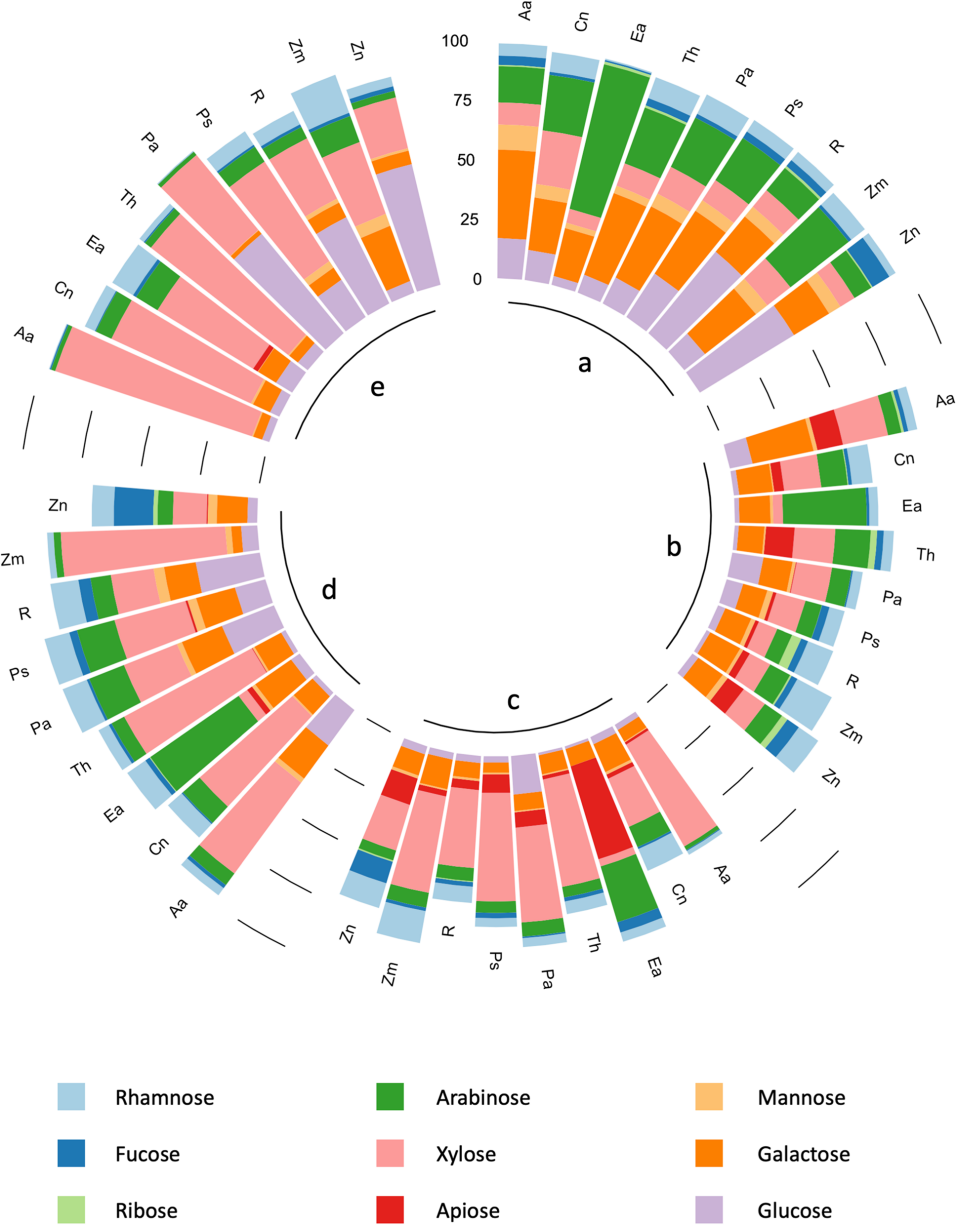
Circular stacked bar plot showing monosaccharide composition of five different fractions after sequential extraction of the different total seagrasses. Solvents: **a**: water, **b**: ammonium oxalate, **c**: hydrochloric acid, **d**: sodium carbonate, **e**: potassium hydroxide. The bar represents neutral monosaccharides + uronic acids = 100%. Therefore, uronic acids make up the blank space up to 100%, thus leading to a negative correlation of the height of the bar and the uronic acid content. For further clarification, detailed values for neutral monosaccharides in % (mol/mol) and uronic acids in % (w/w) are given in Table S3. Aa: *Amphibolis antarctica*, Cn: *Cymodocea nodosa*, Ea: *Enhalus acoroides*, Th: *Thalassia hemprichii*, Pa: *Posidonia australis*, Ps: *Posidonia sinuosa*, R: *Ruppia* sp., Zm: *Zostera marina*, Zn: *Zostera noltii*

A sequential extraction with solvents specific for different polysaccharide classes [38, 39] was performed. An aqueous extraction (solubilizes e.g. arabinogalactans), two pectin-solubilizing solutions (ammonium oxalate and dilute hydrochloric acid) as well as two hemicellulose-solubilizing solutions (sodium carbonate and potassium hydroxide) were used and different yields across fractions (Table S2) were obtained. The monosaccharide composition varied after sequential extractions and among species (Fig. 2; Table S3).

**Fig. 3:**
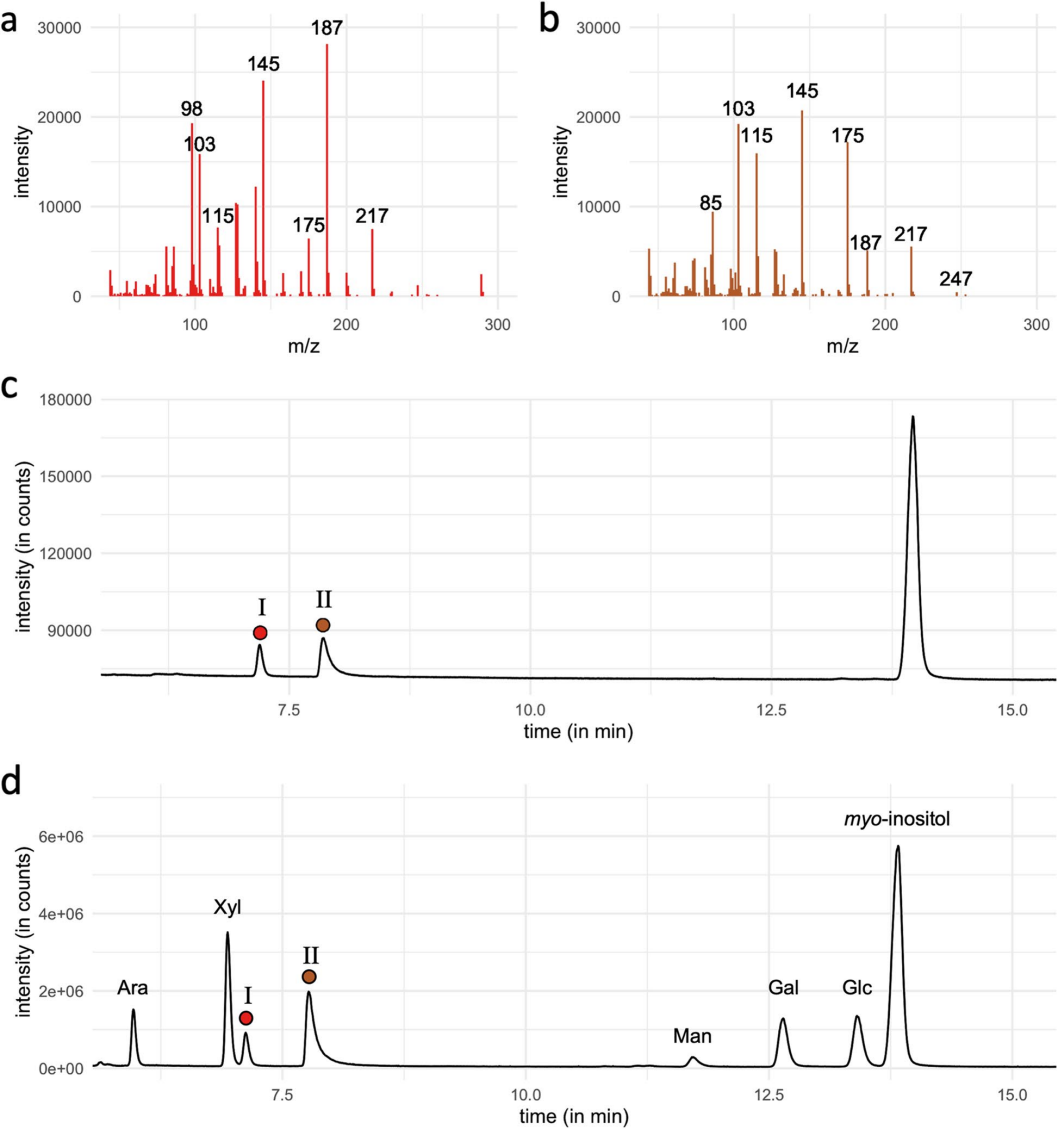
Identification of the unusual constituent monosaccharide apiose. **a**: Electron impact (EI) mass spectrum of the detected apiitol-tetra-acetate. **b**: EI mass spectrum of the detected apiitol-penta-acetate. **c**: Gas chromatogram of the apiose standard with apiitol-tetra-acetate (I) and apiitol-penta-acetate (II). The internal standard *myo*-inositol appears at approximately 13.5﻿ min. **d**: Gas chromatogram of neutral monosaccharides from *E. acoroides* leaves. Apiose peaks are highlighted by colored dots

GC-chromatograms revealed the well-known monosaccharides and a combination of two further peaks (Fig. 3), which were identified by mass spectrometry as two acetylated derivatives (Fig. 3a and 3b) of the unusual branched-chain pentose Api, as confirmed by analysis of a commercial dl-Api standard (Fig. 3c).

The water-soluble fraction a (Fig. 2) showed a strikingly different constituent monosaccharide composition compared to the other fractions with high amounts of Gal and arabinose (Ara, see also Table S3). These neutral monosaccharides are the main components of arabinogalactan-proteins (AGPs), as well as arabinan and galactan side-chains of pectins. They were detected in combined values of approximately 27–85 mol-%. The principal component analysis (PCA, Fig. S1) showed that these two monosaccharides were positively correlated. Preliminary results with β-glucosyl-Yariv reagent (βGlcY), a specific dye for AGPs, supported the presence of AGPs (Pfeifer and Classen, unpublished data).

Fractions b and c isolated by acidic solvents (Fig. 2) represented important similarities, with higher uronic acid and Api contents compared to the other fractions. A PCA-biplot (Fig. S1) underlined this correlation with a low angle of the corresponding loading plots. The ammonium oxalate extract (fraction b) had the highest amounts of uronic acids and the hydrochloric acid extract (fraction c) was richer in xylosyl residues (Xyl).

Finally, the two fractions (d and e) isolated by alkaline solvents (Fig. 2) were characterized by Xyl and glucosyl (Glc) units dominating the monosaccharide composition with two exceptions in the sodium carbonate fraction (fraction d, *E. acoroides* and *Z. noltii*). Dominance of Xyl and Glc was more pronounced in fraction e isolated by potassium hydroxide.

#### Comparison of the carbohydrate compositions of the different species

**Fig. 4:**
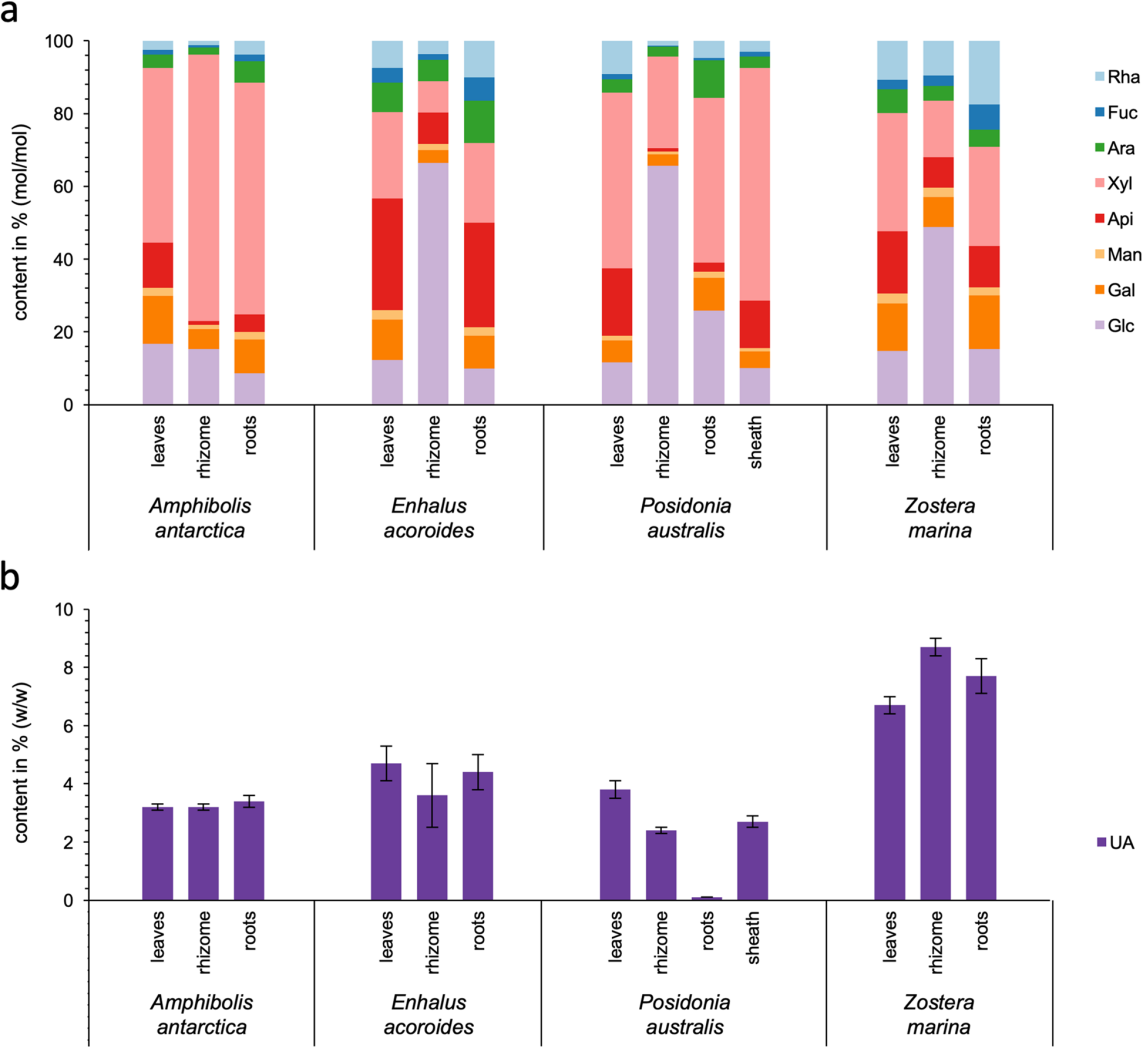
Carbohydrate composition of organs from four different seagrass species (*A. antarctica*, Cymodoceaceae; *E. acoroides*, Hydrocharitaceae; *P. australis*, Posidoniaceae; *Z. marina*, Zosteraceae). **a** Relative neutral monosacccharide composition determined by gas chromatography (GC) (% mol/mol). **b **Absolute content of uronic acids determined by colorimetric assay (% m/m of dry plant weight)

The different families (Posidoniaceae, Zosteraceae, Hydrocharitaceae, Cymodoceaceae and Ruppiaceae) and the different habitats (Baltic, Mediterranean, Red Sea and the Indian Ocean) possess a similar cell wall composition of all nine species in terms of polysaccharides in the different fractions (Fig. 2 and Table S3). Species clearly differed in constituent monosaccharide composition of the different polysaccharides. The dominating monosaccharides Xyl and Glc (possibly xyloglucans) in fraction e show higher amounts of Xyl in the Cymodoceaceae and Hydrocharitaceae contrasting the higher amounts of Glc in the other families. The relative amount of Xyl increased from the aqueous to the potassium hydroxide fraction in all investigated species. Fucosyl (Fuc) residues were only detectable in substantial amounts in *Z. noltii*, especially in the hydrochloric acid and carbonate fractions. *E. acoroides* differed from all other species, with exceptionally high amounts of Ara residues in fractions a-d. This observation was consistent with the results for the different organs (Fig. 4). *E.* *acoroides* was also the only species lacking Api in the ammonium oxalate extract, but presented an extremely high amount (47.5 mol-%) of this unusual monosaccharide in the hydrochloric acid fraction.

### Monosaccharide composition of different organs of four seagrass species

Four representative seagrass species (one of each family) were separated into the different organs (leaves, rhizomes and roots) and analyzed for their constituent monosaccharide composition. (Fig. 4a and b). An additional plant organ – the sheaths at the connection point of leaves and rhizome – was also analyzed in *P. australis*. Neutral monosaccharides with Xyl or Glc residues dominated in all samples, with exception of leaves and roots of *E. acoroides*. Leaves and roots of all species contained more Api compared to the rhizomes, which were rich in Glc.

*A. antarctica* showed the most homogeneous monosaccharide composition of all organs, characterized by high amounts of Xyl residues (between 48.0 and 73.3 mol-% of neutral monosaccharides).

*E. acoroides* presented the highest amounts of Api, especially in leaves (30.6 mol-%) and roots (28.8 mol-%) and also the highest amounts of Ara (up to 11.5 mol-%). The rhizomes were overall much richer in Glc compared to the leaves and roots.

Glc (65.6 mol-%) was the dominating neutral monosaccharide in *P. australis* rhizomes, whereas Xyl was more abundant in leaves, roots and sheaths (45.3–63.9 mol-%). The monosaccharide composition of sheaths, which are remains of dead leaves, resembled that of the leaves. In contrast to all other species, roots of *P. australis* were depleted in uronic acids. All organs of both Australian species, *A. antarctica* and *P. australis*, were enriched in Xyl units compared to *E. acoroides* and *Z. marina*.

Glc was highest in rhizomes compared to the other organs for *E. acoroides*, *P. australis* and *Z. marina*, while Gal and Rha were more abundant in all *Z. marina* organs compared to the other species. Uronic acids (6.7–8.7 % w/w) were significantly more abundant in all *Z. marina* organs compared to the other species (Fig. 4b).

### Microscopical detection of lignin in *Z. marina*

**Fig. 5:**
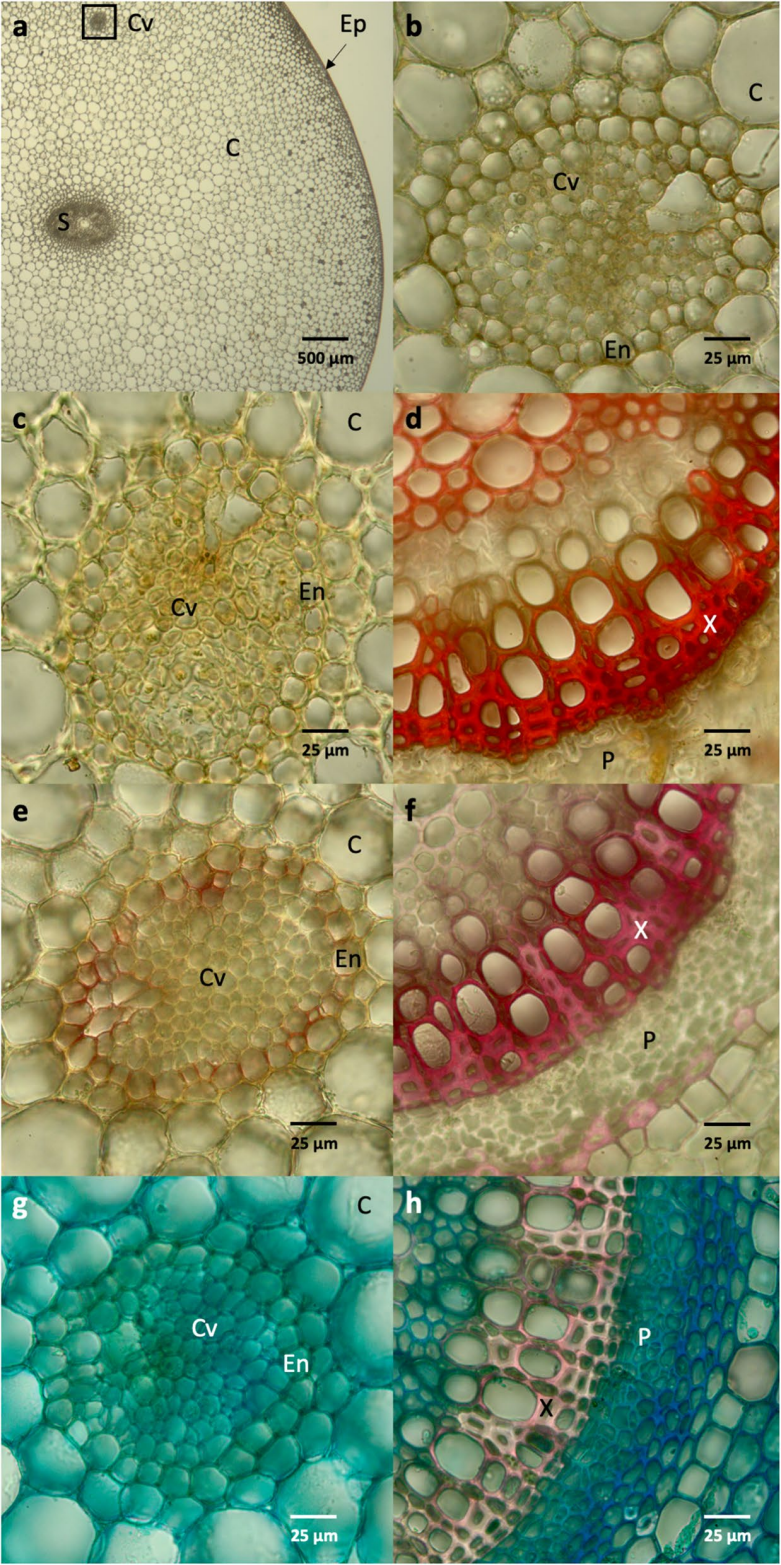
Micrographs of *Z. marina* rhizome and *Mentha* x *piperita* stem sections after staining with different methods to detect lignin. *Zostera*: S: central stele (central cylinder); C: cortex; Cv: cortical vascular bundle; En: endodermis; Ep: Epidermis. *Mentha*: X: xylem; P: phloem. **a**: Unstained cross section of *Z. marina* rhizome with central stele. A small vascular bundle is located within the black frame. **b**: Enlargement of unstained small vascular bundle of *Z. marina*. Three different staining methods for *Z. marina* (left) and *Mentha* x *piperita* with lignified xylem (right). **c** +**d**: Mäule-staining. **e** + **f**: Wiesner-staining. **g** + **h**: Safranin-astra blue staining

Lignified tissues can be detected by different staining methods, each with its own (and different) molecular interaction partner. Staining was performed with cross sections of *Z. marina* rhizomes (Fig. 5a, b, c, e and g) and cross sections of *Mentha* x *piperita* (Lamiaceae) stems as positive control (Fig. 5d, f and h). *Z. marina* rhizome has an epidermis (Ep) and a broad cortex (C) of parenchyma cells with a bigger central stele (S) and two smaller cortical vascular bundles (Cv) [see 40]. Each vascular bundle is surrounded by an endodermis (En). The control shows a part of the vascular bundle of *M. piperita* with Xylem (X) and Phloem (P).

Mäule staining – specifically staining syringyl lignin subunits [40, 41] showed no red color in *Z. marina* (Fig. 5c), whereas the tissue of *M. piperita* (Fig. 5d) showed an intense red dye coloring indicating the presence of lignin, especially in the xylem (X).

Given this S-unit specificity, two additional staining-types were performed. The classical staining method of Wiesner with phloroglucinol/hydrochloric acid, specific for cinnamaldehyde end-groups, but independent of the subunit type, locates the lignin-containing region by formation of red to pink color [40]. The positive control clearly showed the xylem (X) of *M. piperita* in pink (Fig. 5f), but failed to stain *Z. marina* intensively. A slight pink color was detected in some sections mainly in the endodermal region (En; Fig. 5e). Since the Wiesner method, in fact, only detects a very minor substructure of lignin – cinnamaldehyde end-groups are generally very low in abundance in wildtype plants [42], the Wiesner staining might not be sufficiently sensitive to pick up the low levels of lignin expected for *Z. marina*.

Secondly, safranin-astra blue – a differential staining procedure with two dyes – was used to differentiate lignin in light red from cellulose in blue [43]. The staining failed to detect any lignin in the cell walls of *Z. marina* (Fig. 5g), but did develop the expected lignin coloring for *M. piperita* (Fig. 5h). Lignin was expected to be present in at least some of our species, so all samples were further subjected to in-depth analysis by ^13^C-IS pyrolysis-GC–MS, which provides a more accurate and sensitive detection compared to staining techniques.

### Lignin analysis by ^13^C-IS pyrolysis-GC–MS

The different tissues of four selected seagrass species, as representatives of the respective families, were analyzed by quantitative ^13^C-IS pyrolysis-GC–MS, employing a ^13^C lignin isolate as internal standard (^13^C-IS). This selective and sensitive method specifically monitors lignin-derived pyrolysis products to measure total lignin content (and structure), by correlation to the ^13^C-IS lignin [34]. The internal standard used in this analysis was isolated from ^13^C wheat straw [34], and hence, provided an ‘ideal’ standard for herbaceous biomass sources [35]. We are confident that, though the method was optimized for terrestrial grasses, it still allows for a (relative) comparison among seagrasses [35].

**Fig. 6:**
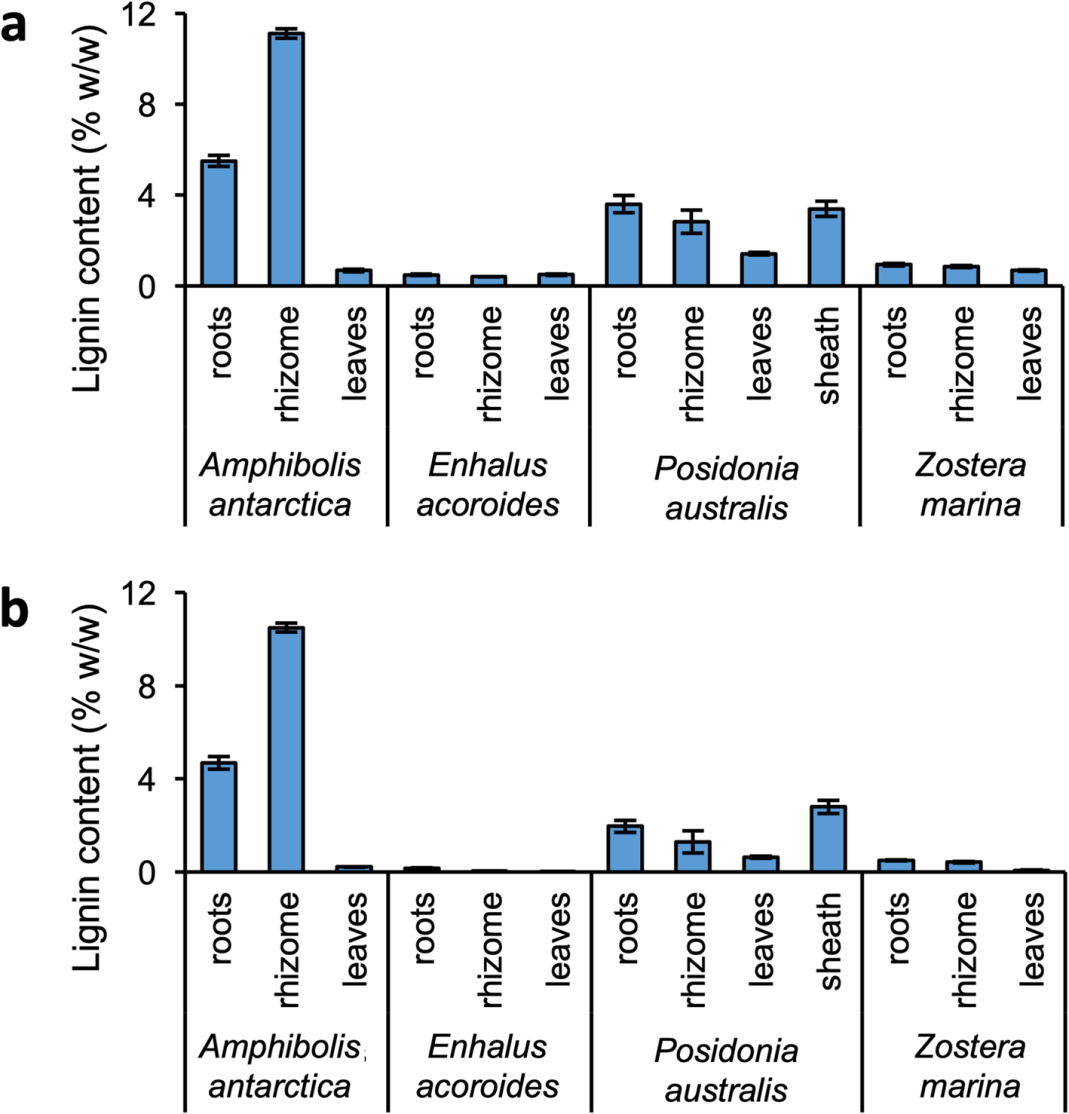
Lignin content as determined by quantitative ^13^C-IS pyrolysis-GC–MS, including ‘all’ lignin-derived pyrolysis products (**a**) and excluding *p*-hydroxyphenyl products, also derived from aromatic amino acids (**b**). Average and standard deviation of technical triplicates

The analysis showed substantial levels of lignin, that varied both by species and tissue type, when ‘all’ *p*-hydroxyphenyl, guaiacyl and syringyl pyrolysis products were included (Fig. 6a). Aromatic amino acids also form *p*-hydroxyphenyl products upon pyrolysis, and if present in considerable amounts, can interfere with the analysis of lignin [34]. Relatively high levels of indole, a marker used to indicate the abundance of aromatic amino acids, and thereby abundance of proteins [44], were detected in the pyrograms (Fig. S2). The indole abundance exceeded that of a reference wheat straw sample by at least a factor of 1.6, confirming that the samples contained relatively high amounts of (interfering) aromatic amino acids compared to that of lignin, especially considering the overall lower lignin contents compared to that of the wheat straw reference (19.4% w/w). Hence, we decided to exclude these *p*-hydroxyphenyl products in the calculations of lignin content, thereby conservatively providing ‘true’ lignin contents for the seagrass samples (Fig. 6b).

The exclusion of *p*-hydroxyphenyl products confirmed the difference in lignin contents among species and organs. The root and rhizome lignin contents in *A. antarctica* (4.7% and 10.5% w/w, respectively) were significantly higher than in the leaves and in the three other species. These tissues were also shown to contain appreciable amounts of ‘true’ lignin in *P. australis*, respectively at 2.0 and 1.3% (w/w). The sheath tissue of *P. australis* was also substantially lignified (2.8% w/w). The roots and rhizomes of *Z. marina* contained very small amounts of lignin (0.5% w/w), in line with previous reports by Klap et al. [29].

The lignified tissues of *A. antarctica* and *P. australis* differed in absolute lignin contents and lignin structure (Table 1). The lignin in *A. antarctica* tissues was found to be richer in syringyl units, with S/G ratios of the roots (~ 0.7) and rhizomes (~ 0.8), respectively, compared to that of *P. australis* (~ 0.2). The sheath tissues of *P. australis* were relatively enriched in syringyl units compared to the other lignified tissues, approximately by a factor two (S/G ~ 0.4).

**Table 1:** Relative abundance of lignin-derived compounds. ^13^C-IS py-GC–MS relative abundance of lignin-derived compounds of the lignified tissues of *A. antarctica* and *P. australis*, excluding *p*-hydroxyphenyl products also derived from aromatic amino acids. Corrected for relative response factors and the relative abundance of ^13^C structural analogues. Summed on the basis of structural classification according to Del Río et al. [45] and Van Erven et al. [46]. Average and standard deviation of technical triplicates

	***Amphibolis antarctica***		***Posidonia australis***		
	roots	rhizome	roots	rhizome	sheath
**Lignin subunits (%)**					
G	59.7 ± 1.0	55.0 ± 0.7	84.0 ± 0.5	84.0 ± 1.9	71.2 ± 3.0
S	40.3 ± 1.0	45.0 ± 0.7	16.0 ± 0.5	16.0 ± 1.9	28.8 ± 3.0
S/G	0.7 ± 0.0	0.8 ± 0.0	0.2 ± 0.0	0.2 ± 0.0	0.4 ± 0.0
**Structural moieties (%)**					
Unsubstituted	10.2 ± 0.4	8.9 ± 0.1	10.9 ± 0.5	10.6 ± 0.9	9.7 ± 0.5
Methyl	5.3 ± 0.1	5.3 ± 0.2	6.6 ± 0.1	6.6 ± 0.1	7.6 ± 0.9
Vinyl	29.6 ± 1.1	24.7 ± 0.3	33.5 ± 0.6	29.9 ± 0.7	30.9 ± 0.7
C_α_-ox	4.8 ± 0.1	5.1 ± 0.0	6.1 ± 0.1	6.0 ± 0.3	7.6 ± 0.1
C_β_-ox^a^	1.6 ± 0.0	1.9 ± 0.0	2.4 ± 0.0	2.7 ± 0.5	4.4 ± 0.3
C_γ_-ox	38.0 ± 2.1	43.2 ± 0.8	30.9 ± 1.0	35.8 ± 3.5	28.1 ± 1.3
Miscellaneous	10.6 ± 0.7	10.9 ± 0.2	9.6 ± 0.2	8.5 ± 1.0	11.6 ± 0.1
PhC_γ_^b^	49.7 ± 1.6	55.9 ± 0.6	41.9 ± 1.1	46.4 ± 2.5	43.4 ± 1.2

^a^excluding diketones. ^b^phenols with intact α,β,γ carbon side chain

A further subdivision of the structural moieties showed that lignins structurally differed beyond the subunit level (Table 1) among organs and species. For the lignin populations of *P. australis* roots and rhizomes this implied that the fine structures presumably still varied in terms of linkage motifs, at least to some extent. The analysis also provided insights into the relative distribution of carbohydrate-derived pyrolysis products, thereby complementing the results obtained by constituent monosaccharide analysis (Fig. S4).

## Discussion

### Sequential extraction of polysaccharide fractions from nine seagrass species

The water-soluble fractions of all seagrass species analyzed here were dominated by Gal and Ara, indicating the presence of arabinogalactans or/and arabinogalactan-proteins. Preliminary results with β-glucosyl-Yariv reagent (βGlcY), a specific dye for AGPs, supported the presence of AGPs in this fraction in all investigated seagrass species (Pfeifer and Classen, unpublished data). Knowledge on AGPs in seagrass cell walls is limited. Pettitt (1980) showed that pollen of *T. hemprichii* were stained by βGlcY, [47]. Recently, isolation and characterization of an AGP from a seagrass was successfully performed and revealed unusual features such as a high degree of branching and a high content of terminating 4-*O*-methyl-glucuronic acid (4-*O*Me GlcA) residues [48]. Our results show, for the first time, that occurrence of AGPs seems to be a common feature of seagrass species from all families and all habitats.

The constituent monosaccharide composition of the ammonium-oxalate fraction with uronic acids, Api, Gal, Ara, Xyl and Rha showed that pectic polysaccharides are present, as expected for this type of extracts. The group of pectins include the major polysaccharides homogalacturonan (HG), rhamnogalacturonan I (RG-I), rhamnogalacturonan II (RG-II) and xylogalacturonan (XGA) (for review see [49, 50]). Up to now, investigations on *Zostera* cell walls revealed evidence for presence of HG [51, 52] and RG-I [21, 51]. The presence of Rha in the fractions isolated with ammonium oxalate and hydrochloric acid suggests that RG I is also present in the cell walls of the seagrasses investigated here. All investigated seagrass species are distinct from terrestrial flowering plants as they contained substantially higher amounts of Api, most likely derived from apiogalacturonan (AGA). The Lemnoidae (subfamily of the Araceae), which are also non-commelinid monocots and members of the order Alismatales, are the only other flowering plants known to have cell walls with AGA [53, 54]. This special pectic polysaccharide has a (1 → 4)-linked α-d-GalA backbone that is substituted at *O*-2 with single Api*f* residues or short oligosaccharides of Api*f.*

AGA in seagrasses was first isolated from *Zostera* [55], further studied [56] and structurally characterized [21, 52]. High contents of Api have also been found in *P. australis* [57] and *Heterozostera tasmanica* [58]. There are intergeneric differences in the composition of pectin subclasses in the duckweeds, especially in the group of substituted HGs. AGAs are dominant in the ammonium oxalate fraction of *Spirodela*, *Landoltia* and *Lemna*, whereas *Wollfiella* species contain mainly Ara and in the latest diverging genus *Wolffia*, XGAs are most abundant [53]. Interestingly, RG-II, which is strongly conserved in the plant kingdom [59] and also present in the aquatic Lemnoideae [53], also contains small amounts of Api, but has not been described in any seagrass. The presence of AGA- and RG-II domains has been proposed in the lemnoids [53]. A typical feature of RG-II is presence as borate cross-linked dimer. *Lemna* cell walls have been reported to contain high amounts of boron, but only a small portion is associated with RG-II [60]. Hence it has been proposed, that most of this boron is derived from borate esterified AGAs [53]. This suggests the isolation and characterization of AGA and RG-II from cell walls of seagrasses to be a productive path for future research.

The seagrass fractions isolated by diluted hydrochloric acid were probably also pectin-rich, with the difference that Xyl content was higher compared to the ammonium oxalate fractions. One reason might be the presence of XGAs, which are also known from the Lemnoideae. Members of the most recently evolved genus in this subfamily, *Wolffia*, show a higher prevalence of XGA than AGA [53].

The monosaccharide composition in both fractions soluble in acidic solvents was very different in *E. acoroides*. It is the only species which contained no Api in the ammonium oxalate extract, but an extremely high amount (nearly 50%) of this monosaccharide in the hydrochloric acid fraction, indicating differences in the fine structure of the AGAs with different solubility. Furthermore, exceptional high amounts of Ara in both fractions of *E. acoroides* are striking. It seems unlikely, that these amounts are present only in side chains of RGs. Another possibility would be that singular arabinans are abundant in this species. Arabinans may occur separately or as side chains of rhamnogalacturonan I and are composed of a backbone of α-(1 → 5)-linked Ara*f* units, which can be substituted at positions *O*2, *O*3, and both *O*2 and *O*3 with monomeric or oligomeric Ara side chains [61]. Cell walls of the genus *Wolffiella* (Lemnoideae) are also characterized by a high Ara content [53]. The littoral species *E. acoroides* belongs to the Hydrocharitaceae, a family which includes freshwater and marine species. It is the only seagrass species which does not complete its life cycle entirely under water [2]. *E. acoroides* is dioecious, with male flowers dehiscing and floating on the water surface to pollinate female flowers: the pollen and the styles remain dry [62].

Xyl or Glc are dominating in the fractions isolated by sodium carbonate, with exception of *E. acoroides*, where again Ara is the main monosaccharide with over 50 mol-%, and *Z. noltii* with high Fuc content (see below). Xyl and Glc are typical monosaccharides of plant hemicelluloses, especially xylans and xyloglucans and in some species also mixed-linked glucans. Xylans are major components of secondary cell walls in terrestrial plants. They have a backbone consisting of (1 → 4)-linked β-d-Xyl*p* with a high degree of substitution with Ara*f* (sometimes substituted with ferulic acid) and/or (4*O*Me)-GlcA [63–65]. Side chains containing Ara*p* are most abundant in the arabinopyranosyl-containing glucuronoxylans of the Alismatales [63] and might therefore also be present in seagrass cell walls. Xylans have been detected in cell walls of *Halophila* species [66] and *Z. marina* [17], although the exact structure of seagrass xylans is unknown. Xyloglucans consist of (1 → 4)-linked β-d-Glc which are substituted with α-d-Xyl chains at *O*-6. Fucogalactoxyloglucans are common in walls of non-commelinid monocotyledons and might occur especially in the carbonate fraction of *Z. noltii* with high Fuc content. Fuc was not detected in the same fraction of *Z. marina* in this study. In a study with the monoclonal antibody CCRCM1, which recognizes the epitope structure α-Fuc-(1,2)-β-Gal of fucogalactoxyloglucans, labelling of *Zostera muelleri* was weak and restricted to the phloem sieve elements [67].

In the fractions isolated by potassium hydroxide, dominance of Xyl and Glc was striking. Xyl was present in very high amounts in the Hydrocharitaceae and the Cymodoceaceae (between 53.2 and 89.4 mol-%). Xyl was less pronounced (between 24.4 and 53.6 mol-%) in the Posidoniaceae, Ruppiaceae and Zosteraceae and accompanied by appreciable amounts of Glc. Further investigations of xylans and xyloglucans of seagrasses are required to elucidate the fine structures of these polysaccharides in these marine organisms.

### Constituent monosaccharide composition of different organs of four seagrass species

The monosaccharide composition of all organs in *A. antarctica* was quite homogeneous and characterized by high amounts of Xyl (48.0—73.3 mol-%). The organs of all other species differed in their compositions. The rhizomes of *E. acoroides*, *P. australis* and *Z. marina* were characterized by higher amounts of Glc (48.8—66.4 mol-%) compared to leaves and roots, and in case of *P. australis* also sheaths. Rhizomes are persistent and the main storage organs for soluble carbohydrate reserves. Sucrose has been identified in most seagrasses as main carbohydrate storage molecule, while also starch is found [68]. Starch supports the storage function in aboveground tissues and sucrose in belowground plant parts [69]. The rhizome of *A. antarctica* is very thin and therefore not capable of storing as much carbohydrate reserve as both *Posidonia* species. Xyl residues are more abundant in *A. antarctica* and *P. australis*, a feature which might be correlated to the occurrence of lignin.

### Lignin in different seagrasses

Our results highlight the sensitivity of the ^13^C-IS pyrolysis-GC–MS technique for evaluating lignin contents, and given this sensitivity our analyses show that *E. acoroides* contains a negligible amount of lignin. Likewise, leaves contained negligible amounts of lignin in all species, except those of *P. australis* [25–28], which showed approximately 0.6% (w/w) lignin.

The lignin contents for *P. australis* were substantially lower than those reported by Kaal et al. [36] for *P. oceanica*. First and foremost, the gravimetric method used previously to determine lignin [36] can overestimate lignin contents due to its low selectivity, and is expected to include considerable amounts of ash, protein, and tannin, amongst other non-extractable phenolic oligomers and polymers [33, 70]. Indeed, substantial tannin/polyphenol-derived pyrolysis products (e.g., catechol, methylcatechol, methoxycatechol and gallic acid) were also detected in our samples (Fig. S3). Moreover, Kaal et al. [36] prepared extractive-free cell wall residues, while we measured the samples in their entireties, which further contributes to the relatively lower lignin contents reported here.

Lignin is commonly considered to provide plants with mechanical strength and rigidity, i.e. allowing plants to grow upward, which logically is a feature of lesser relevance in an underwater environment [71]. Tougher plants might still be better protected against stronger currents and, hence, the presence of lignin might be an evolutionary advantage in this context. Lignin is known to provide protection against pathogenic microbes [72], which may benefit seagrass [73]. In contrast to that, retention of lignin might also be understood as redundant artifact of their terrestrial ancestors. Our results provide evidence for differential seagrass evolution relative to the lignification of their cell walls. Genetic engineering of the seagrasses *A. antarctica* and *P. australis*, primarily targeted to modify their lignin biosynthetic pathways, might be of help for answering these questions [74].

The presence of lignin correlated well with the abundance of Xyl residues, which presumably is a measure of xylan abundance in these organs and, hence, could indicate that some general features of terrestrial secondary cell walls have been conserved in some seagrass species. Xyl residues in terrestrial grasses are mainly part of glucuronoarabinoxylans, which are involved in the covalent cross-linking to lignin via diferulic acid moieties to form so-called lignin-carbohydrate complexes [64]. Indeed, substantial amounts of ferulic acid have been detected in *P. oceanica* lignins [37].

### Adaptation of seagrasses to the marine environment

Cell walls of marine algae are characterized by polysaccharides with high charge, e.g. the polyanionic alginates and fucose-containing sulfated polysaccharides of the brown algae [75] or the sulfated galactans of the red algae [76]. Sulfated galactans also occur in cell walls of some green algae (e.g. [77]) and have been detected in the seagrasses *C. nodosa*, *Halodule wrightii*, *Halophila decipiens* and in the facultative marine angiosperm *Ruppia maritima* [18–20]. The amount of this sulfated galactan in *Ruppia* increased with salinity and disappeared in culture without salt supplement [78].

Additional components of seagrass cell walls might be involved in adaptation to the marine environment. It has been shown that salt stress upregulates periplasmic arabinogalactan proteins [79]. These glycoproteins have been isolated from *Z. marina* and are characterized by a high content of glucuronic acid (GlcA) residues in general and terminal 4-*O*Me-GlcA in particular [48]. Structure-dependent calcium-binding by AGPs could putatively help the plant to discriminate against binding of Na^+^ [80]. Comparison of transcriptomic data for *Zostera* grown with and without NaCl revealed that genes involved in calcium-signaling were upregulated after NaCl treatment [81]. Interestingly, the amount of AGPs in roots and rhizomes of *Z. marina* was around 20 fold higher than in leaves [48], maybe because rhizomes and roots live longer and need increased responsive capacity via signaling AGPs. In the marine angiosperm *Ruppia* sulfated galactans possibly involved in salt protection are also mainly present in the rhizomes [18]. Arabinans are also known to be involved in salt stress tolerance [82–84]. *E. acoroides* was very rich in Ara, so in this species arabinans may be important to cope with high salinity.

The most prominent feature of pectins is their uronic acid rich, hence highly charged, structure. We have shown that all seagrasses investigated in this study independent of family assignment or habitat possess high Api contents. This is an important hint for presence of the unusual pectic AGAs, possibly accompanied by other pectic polysaccharides. AGAs have a very low degree of esterification around 10% [85] and possess stronger ability to bind cations by non-methyl esterified galacturonosyl residues. An increase in the number of pectin methylesterase genes detected in the genomes of two *Zostera* species seems to be responsible for the low degree of methyl esterification and was understood as putative control mechanism for osmoregulation [13, 14]. In all seagrass species, which we separated into the organs (one of each family), Api was most abundant in leaves, which might be a hint towards different mechanisms to cope with salt stress. These could be differentially realized in the separate organs. More studies are needed on occurrence and structures of sulfated galactans, AGPs and pectic polysaccharides of seagrasses to elucidate how special features of seagrass cell walls contributed to one of the most extreme evolutionary events witnessed in the angiosperm lineage.

### Evolutionary implications

Seagrasses belong to the monocotyledonous order of Alismatales, a basal group in flowering plants [5]. The fossil record for seagrasses is limited and patchy, but indicates that ancestors of modern seagrasses evolved successfully in the Tethys Sea during the late Cretaceous [86].

Evolutionary pressures have likely constrained divergence within the group, and in fact, Cretaceous to Miocene aged *Posidonia* fossils have remained apparently almost unchanged over this long evolutionary history [87]. Molecular data show that seagrasses emerged from a freshwater ancestor of terrestrial origin through at least three independent ‘return to the sea’ events [2]. A combination of low sequence divergence rates (e.g. [88, 89]) and clonal growth is consistent with a low number of species. In contrast to that, some authors understand the lack of speciation amongst seagrasses as a hint for major extinctions throughout their evolutionary history [86]. Our findings on saccharides present in seagrass cell walls revealed similarities among all species, most prominent the putative occurrence of AGA. AGA is taxonomically restricted to the Alismatales, with Lemnoidae (subfamily of the Araceae) being the only other flowering plants known to have cell walls containing AGA [53, 54]. Our finding of high amounts of Api in all investigated seagrass species, representing the three independent lineages, suggests the unusual pectic polysaccharide AGA was already present in terrestrial ancestors and not the result of convergence among independent seagrass lineages.

During diversification of the Lemnoideae from lemnoids (*Spirodela, Landoltia* and *Lemna*) to wolffioids (*Wolffiella* and *Wolffia*), AGA decreased substantially [53, 90]. Amounts of Api in seagrass cell walls are more similar to lemnoid species than to wolffioids. In *Wolfiella* species, high amounts of Ara are present, a feature we also detected in *E. acoroides* cell walls. *E. acoroides* is also outstanding from an ecological perspective as it is the only seagrass that is not hydrophilous.

Further differences comprise putative xylans and/or xyloglucans in the fractions isolated by potassium hydroxide. In the Hydrocharitaceae and the Cymodoceaceae, Xyl is dominating with up to 90% (more xylans?), whereas in the Posidoniaceae, Ruppiaceae and Zosteraceae, Xyl is less prominent and accompanied by appreciable amounts of Glc (more xyloglucans?).

Biosynthesis of lignin has been conserved in the seagrass species *A. antarctica* and *P. australis*. Roots and rhizomes of *A. antarctica* and in case of *P. australis* also sheaths contain lignin in amounts comparable to duckweed species [90]. *E. acoroides* and *Z. marina* are nearly lignin-free. Probably, the biosynthetic lignin pathway has not been lost in some species, but transcription might be regulated with regard to environmental needs. For example, *A. antarctica*, which has tough, wiry rhizomes and stems that grow up to 2 m high (in elevated salinities in Shark Bay), had the highest lignin content in roots and rhizomes.

Comparisons with cell walls of freshwater members of the Alismatales will be necessary to distinguish between more general adaptations to the aquatic environment and those specific for the marine environment. The elucidation of fine structures of cell wall components will also help to understand the evolutionary origins of seagrasses.

## Conclusions

Despite the ecological importance of seagrasses, which form important coastal ecosystems worldwide, seagrass cell walls, as a whole, are poorly understood. Comparisons of a greater number of species in different habitats are needed. Our investigations show that all nine species from the different families probably possess similar polysaccharides like arabinogalactans, AGAs, xylans and xyloglucans. High amounts of Api, which is part of the unusual AGAs known from duckweeds and some seagrass species (especially *Z. marina*) have been detected in all investigated seagrass species and AGA might therefore be regarded as common across all seagrasses and possibly their evolutionary ancestors.

However, significant quantitative differences in the monosaccharide compositions were observed among species and plant organs. *E. acoroides* is outstanding with highest amounts of Api, especially in the fraction isolated by hydrochloric acid, and highest amounts of Ara. Another striking difference of the cell wall compositions are highly variable amounts of lignin. *A. antarctica* and *P. australis* contain appreciable amounts of lignin, especially in rhizomes and roots and in case of *P. australis* also in sheaths. *Z. marina* and *E. acoroides* are nearly free of lignin. Lignin-free cell walls could provide a very interesting raw material for sustainable production of biofuels and materials. Furthermore, the lignified tissues of *A. antarctica* and *P. australis* differed considerably in terms of lignin structure, the lignin of *A. antarctica* being richer in syringyl units compared to that of *P. australis*. The retention of lignin during evolution from terrestrial vascular plants back to the sea might be seen as a redundant artifact or an evolutionary advantage in marine environments. The lignified rhizomes of *A. antarctica* and *P. australis* might indeed provide strength for anchoring required to grow in areas with strong tidal movement and highly exposed, swell/dominated coastlines.

Our investigations allow comparative insights into the general composition of seagrass cell walls and adaptive cell wall modifications of angiosperms to survive in the marine environment. The comparison of nine species offers information on identical or different strategies of seagrasses with regard to the challenging evolutionary step to life in saltwater. Although the composition of cell walls varied among species and lineages, the sharing of unusual AGAs among seagrasses and Lemnoids suggest some traits predate all ‘Return to Sea’ events. Further characterization of seagrass polysaccharide and lignin structures, as well as whole genome sequencing of more seagrass species are necessary to understand the evolution of this special group of angiosperms and to adequately protect coastal ecosystems.

## Materials and Methods

### Seagrass material

Seagrass species native to the Baltic Sea were collected in Kiel-Schilksee (*Zostera marina*) and Neustadt in Holstein (*Ruppia* spp. and *Zostera noltii*).

Other seagrass species were collected and kindly provided by different collaboration partners from Australia (*Amphibolis antarctica*, *Posidonia australis* and *Posidonia sinuosa*, Dr. Elizabeth A. Sinclair, University of Western Australia), Indonesia (*Enhalus acoroides,* Meri Yulvianti, Kiel University, Germany), Malta (*Cymodocea nodosa,* Prof. Dr. Joseph A. Borg, University of Malta) and Saudi-Arabia (*Enhalus acoroides* and *Thalassia hemprichii*, Prof. Dr. Carlos M. Duarte, KAUST, Saudi-Arabia). A detailed list of the relevant collection data is provided in Supplementary Table S1.

All seagrass plant material was collected, rinsed in tap water and subsequently dried by a mild drying method (freeze-drying or oven-drying below 60 °C).

### Sequential extraction of polysaccharides

Freeze dried seagrass samples (bulk material) of the 9 species were prepared in an IKA MF 10 basic laboratory grinder (sieve size: 1.0 mm; IKA-Werke GmbH & Co.KG, Staufen, Germany). Milled samples were extracted four times with aceton:water (70:30) in a ratio of 1:10 (w/v) overnight under constant shaking on a laboratory shaker (Edmund Bühler GmbH, Bodelshausen, Germany) at 4 °C. The extract was separated by vacuum filtration. An aqueous extraction of the insoluble residue was performed afterwards with double-distilled water under the same conditions. The extract was separated by vacuum filtration and the insoluble residue was subsequentially treated with 0.2 M ammonium oxalate (Carl Roth GmbH & Co.KG, Karlsruhe, Germany), 0.1 M hydrochloric acid (diluted from 37%, p.a., Carl Roth GmbH & Co.KG, Karlsruhe, Germany), 3% (w/v) sodium carbonate (Merck KGaA, Darmstadt, Germany) and 2 M potassium hydroxide (Honeywell Inc., Morristown, NJ, USA). For each step, the insoluble residue was treated with the respective solution in a ratio of 1:100 (w/v) under constant stirring at 70 °C for 21 h. Each extract was separated from the insoluble residue by centrifugation (Hereus Multifuge X3, Thermo Fisher Scientific Corp., Waltham, MA, USA) at 19.000 g for 30 min. All extracts were dialyzed for 3 days against demineralized water (MWCO 12–14 kDa, Medicell Membranes Ltd., London, UK), except for the aqueous and the sodium carbonate extract, which were precipitated with absolute ethanol or acetone, respectively, in a final concentration of 80% (v/v). After cooling overnight at 4 °C the precipitate was centrifuged at 4122 g, re-dissolved in demineralized water and freeze-dried (Christ Alpha 1–4 LSC, Martin Christ GmbH, Osterode, Germany). The extraction procedure was done once from the bulk material. A detailed visualized extraction protocol is also provided in the Supporting information of Pfeifer et al. [91].

### Neutral monosaccharide composition

Monosaccharide composition was determined following the acetylation protocol of Blakeney et al. [92], with a modified hydrolysis method. 1–10 mg sample were hydrolysed in 2 M trifluoroacetic acid for 1 h at 120 °C (Bioblock Scientific, Thermolyne Corp., Ramsey, MN, USA). 0.5 mg of *myo*-inositol was included as internal standard. For the crude plant samples, 15–20 mg were used and particles remaining after hydrolysis (e.g. cellulose) were removed by cotton wool filtration. The resulting dichloromethane layer was analysed via GC (Agilent 7890B, Agilent Technologies, Inc., Santa Clara, CA, USA; column: Optima-225, 25 m, 0.25 mm, 0.25 µm; flow rate: 1 mL/min; temperature 230 °C; split ratio 30:1). The acetylated monosaccharides were determined by GC-FID via retention time with verification through a parallel coupled mass spectrometer (Agilent 5977B MSD, Agilent Technologies, Inc.). Measurements were performed as triplicates, meaning here three independent derivatization experiments with subsequent GC-analysis. Apiose was additionally verified by comparison with a commercially purchased standard (DL-apiose, Carbosynth Ltd., Berkshire, UK). A Principal Component Analysis (PCA) was performed to compare overall monosaccharide composition using RStudio software (version 1.2.5042) with the “ggfortify” package for calculation and biplot visualization [93].

### Colorimetric quantification of uronic acids

The content of uronic acids was determined photometrically (UVmini-1240, Shimadzu AG, Kyoto, Japan) by a modified procedure of Blumenkrantz and Asboe-Hansen [94]. 1 mg of polysaccharide sample was dissolved in 2 mL of sulphuric acid (4% v/v, prepared from concentrated sulfuric acid p.a., Rotipuran, Carl Roth GmbH & Co.KG, Karlsruhe, Germany). A hydrolysis step was performed on crude plant samples using diluted sulphuric acid at 120 °C for 60 min. These solutions were also standardized to a concentration of 1 mg/2 mL. Three measurements (technical replicates) were done and an additional measurement without colouring reagent for each sample was treated as background.

### Staining of lignin and microscopical evaluation in *Z. marina*

Three classical staining methods were used for microscopic evaluation of lignin content in *Z. marina* rhizome. *Mentha* x *piperita* stems (collected from the botanical garden of the Pharmaceutical Institute, Kiel University) were used as a positive control for lignin-containing tissue in all three staining methods. Fresh *Z. marina* rhizome was collected (Kiel-Schilksee) and cut with a hand-microtome (Euromex Microscopen BV, Arnhem, The Netherlands) into sections of a single cell-layer thick. These were stored in 80% ethanol until use.

Firstly, Mäule staining (using the protocol of [40]) was performed by applying 0.5% potassium permanganate solution (prepared from KMnO_4_, ≥ 99%, Ph.Eur., Carl Roth GmbH & Co.KG, Karlsruhe, Germany) to the microscopic sections in a 2.0 mL microtube with a following incubation time of 2 min. The permanganate solution was then removed from the microtube and the sections were rinsed with double-distilled water until the solution stayed clear. The water was removed and 3% HCl (37%, p.a., Carl Roth GmbH & Co.KG, Karlsruhe, Germany) was applied two times for about 5 min each until the brown color disappeared. Concentrated ammonium hydroxide was then pipetted into the microtube. The section was investigated under the microscope (Carl Zeiss AG, Oberkochen, Germany).

Secondly, Wiesner staining according to the protocol of [40] was used. Two volumes of a solution of 3% (m/v) phloroglucinol (synthesis grade, Merck KGaA, Darmstadt, Germany) in ethanol (≥ 99.8%, p.a., Carl Roth GmbH & Co.KG, Karlsruhe, Germany) was combined with one volume of concentrated HCl (37%, p.a., Carl Roth GmbH & Co.KG, Karlsruhe, Germany). This solution was transferred onto the slices in a microtube and incubated at room temperature for 1 min. The sections were then investigated microscopically with lignin-containing cells appearing dark pink.

Thirdly, a safranin-astra blue differential staining (modified from [95]) was utilized. A 1% (m/v) safranin (Merck KGaA, Darmstadt, Germany) solution in double-distilled water and a 0.5% (m/v) astra blue (Merck KGaA, Darmstadt, Germany) solution in 2% tartaric acid (Merck KGaA, Darmstadt, Germany) were made. The astra blue solution was added to the sections in a microtube and incubated for 5 min, followed by a washing step with double-distilled water. Afterwards, the safranin solution was applied for 5 min with a subsequent differentiation step in 70% ethanol. The slices were washed with acidified 70% ethanol until clear red and blue appearance was visible. All micrographs were obtained by a Canon EOS 1000D (Canon AG, Tokyo, Japan).

### Quantitative pyrolysis-GC–MS with ^13^C lignin as internal standard

Analytical pyrolysis coupled to gas chromatography with high-resolution mass spectrometric detection (Exactive Orbitrap, Thermo Scientific, Waltham, MA, USA) was performed as previously described [35].

Uniformly ^13^C-labeled lignin (97.7 atom% ^13^C), isolated from ^13^C wheat straw (Iso*Life* BV, Wageningen, The Netherlands) was used as an internal standard (^13^C-IS) [34, 35]. To each sample (80 µg), 10 µL of a ^13^C-IS solution (1 mg/mL ethanol/chloroform 50:50 v/v) was added and dried prior to analysis [34]. All samples were prepared and analyzed in triplicate (technical replicates). Lignin-derived pyrolysis products were monitored in full MS mode on the most abundant fragment per compound (both nonlabeled and uniformly ^13^C labeled). Pyrograms were processed by TraceFinder 4.0 software. Lignin contents and relative abundances of lignin-derived pyrolysis products were calculated, as described previously [35]. Indole, catechol, methylcatechol, methoxycatechol and gallic acid and various carbohydrate-derived pyrolysis products were also monitored in full MS mode on the most abundant fragment per compound, but conversely to lignin without applying response correction.

## Supplementary Information

**Additional file 1. **

## Data Availability

The datasets used and/or analysed during the current study are available from the corresponding author on reasonable request.
