# Supplementary material for: Profiling the cell walls of seagrasses from A (Amphibolis) to Z (Zostera)
Source: BMC Plant Biol. 2022 Feb 4;22:63. doi: 10.1186/s12870-022-03447-6 (PMC8815203; doi:10.1186/s12870-022-03447-6)
Supplement: Supplementary file 1 — Additional file 1. [file 12870_2022_3447_MOESM1_ESM.pdf]

Supplement: Profiling the Cell Walls of Seagrasses from A (*Amphibolis*) to Z (*Zostera*)

Lukas Pfeifer [1], Gijs van Erven [2], Elizabeth A. Sinclair [3], Carlos M. Duarte [4], Mirjam A. Kabel [2] and Birgit Classen [1]

[1] Pharmaceutical Institute, Department of Pharmaceutical Biology, Christian-Albrechts-University of Kiel, Gutenbergstr. 76, 24118 Kiel, Germany

[2] Laboratory of Food Chemistry, Wageningen University & Research, Bornse Weilanden 9, 6708 WG, Wageningen, The Netherlands

[3] School of Biological Sciences and Oceans Institute, University of Western Australia, Crawley, WA, Australia

[4] Red Sea Research Center (RSRC), King Abdullah Univerity of Science and Technology, Thuwal, Saudi Arabia

e-mail-addresses: lpfeifer@pharmazie.uni-kiel.de, gijs.vanerven@wur.nl, elizabeth.Sinclair@uwa.edu.au, carlos.duarte@kaust.edu.sa, mirjam.kabel@wur.nl, bclassen@pharmazie.uni-kiel.de

[*] Corresponding author:

Birgit Classen, Pharmaceutical Institute, Department of Pharmaceutical Biology, Christian-Albrechts-University of Kiel, Gutenbergstr. 76, 24118 Kiel, Germany

Phone: +49-431-8801130

Fax: +49-431-8801102

e-mail: bclassen@pharmazie.uni-kiel.de

Table S1:

Detailed list of seagrass origin and collection information

**Family**	**Species**	**Collector**	**Location**
Cymodoceaceae	*Cymodocea nodosa* (Ucria) Asch.	Prof. Dr. Joseph A. Borg, Andrew Agius; University of Malta	Qualet Marku, Malta
	Amphibolis antarctica (Labill.) Asch.	Dr. Elizabeth Sinclair; University of Western Australia	25.85324 S 113.75616 E Shark Bay, Australia
Hydrocharitaceae	*Enhalus acoroides* (L.f.) Royle	Prof. Dr. Carlos Manuel Duarte; King Abdullah University of Science and Technology (KAUST)	22.390989 N, 39.135561 E Saudi-Arabia
	Enhalus acoroides (L.f.) Royle	Meri Yulvianti; Kiel University	6.620583 S, 110.637585 E, Indonesia
	Thalassia hemprichii (Ehrenb. ex Solms) Asch.	Prof. Dr. Carlos Manuel Duarte; KAUST	22.394761 N, 39.131512 E, Saudi-Arabia
Posidoniaceae	*Posidonia australis* Hook.f	Dr. Elizabeth Sinclair; University of Western Australia	25.85282 S 113.76023 E Shark Bay, Western Australia
	Posidonia sinuosa Cambridge et Kuo	Dr. John Statton; University of Western Australia	32°16'19.01" S 115°41'25.65" E Point Peron, Western Australia
Ruppiaceae	*Ruppia* spp.	Lukas Pfeifer; Kiel University	54.088582 N, 10.801559 E, Germany
Zosteraceae	*Zostera marina* L.	Lukas Pfeifer; Kiel University	54.427500 N, 10.171528 E, Germany
	Zostera noltii Hornem.	Lukas Pfeifer; Kiel University	54.088582 N, 10.801559 E, Germany

Table S2:

Dry matter yields from the nine sequential extractions fractions from the nine seagrass species. Values are given in % (w/w).

Plant	Aqueous	Ammonium oxalate	Hydrochloric acid	Sodium carbonate	Potassium hydroxide	Residue[a]
A. antarctica	2.1	8.0	4.2	3.5	5.8	76.3
C. nodosa	1.9	11.4	4.7	7.5	8.7	65.7
E. acoroides	2.5	4.3	9.7	5.3	9.1	69.2
P. australis	1.4	5.7	5.6	4.0	12.2	71.1
P. sinuosa	2.0	4.8	3.2	2.9	5.6	81.5
R. spp.	2.3	10.8	8.5	3.8	12.4	62.3
T. hemprichii	2.0	5.9	4.6	8.6	12.9	66.0
Z. marina	2.7	5.1	4.4	1.5	3.3	83.0
Z. noltii	2.5	9.7	5.0	2.8	18.0	62.0

[a]Calculated residual yield based on 100 minus yields in extracts (% (w/w)).

Table S3:

Constituent monosaccharide composition of different polysaccharide fractions obtained by sequential extraction. Neutral monosaccharide values are given in % (mol/mol) and uronic acids in % (w/w). In brackets, the standard deviation of three derivatization experiments with subsequent GC-analysis is given.

**Plant**	**Extr.-ID**	**Plant**	**Rha**	**Fuc**	**Rib**	**Ara**	**Xyl**	**Api**	**Man**	**Gal**	**Glc**	**Uronic acids**
Aqueous extract	1	A.a.	5.2 (±0.3)	3.9 (±0.2)	0.6 (±1.0)	15.4 (±2.5)	9.4 (±0.9)	-	10.7 (±0.9)	37.7 (±3.7)	17.1 (±6.1)	1.7
	2	C.n.	8.7 (±0.7)	1.4 (±0.2)	-	24.3 (±2.3)	23.5 (±1.5)	-	5.9 (±0.5)	23.0 (±0.5)	13.2 (±2.2)	3.9
	3	E.a.	0.6 (±0.2)	0.7 (±0.2)	1.2 (±0.1)	63.9 (±1.2)	6.1 (±0.2)	-	2.4 (±0.2)	21.2 (±1.0)	3.9 (±1.1)	2.7
	4	P.a.	9.3 (±1.6)	1.8 (±1.8)	-	23.6 (±1.2)	12.8 (±0.7)	-	6.0 (±0.4)	34.1 (±2.9)	12.4 (±1.6)	2.3
	5	P.s.	5.8 (±0.4)	3.2 (±0.2)	-	21.5 (±1.0)	10.2 (±0.2)	-	5.3 (±0.5)	32.0 (±1.2)	22.0 (±0.8)	1.7
	6	R.	4.5 (±0.7)	3.3 (±0.2)	1.0 (±0.1)	12.3 (±1.5)	9.1 (±0.8)	-	5.3 (±0.4)	19.5 (±2.0)	45.0 (±4.6)	1.6
	7	T.h.	9.7 (±1.5)	3.4 (±0.5)	1.0 (±1.7)	25.9 (±1.5)	10.4 (±0.9)	-	3.4 (±1.2)	37.9 (±1.8)	8.3 (±1.9)	4.1
	8	Z.m.	7.5 (±0.1)	1.5 (±0.8)	-	32.3 (±1.9)	12.8 (±0.9)	-	6.2 (±0.5)	28.3 (±0.7)	11.4 (±0.5)	4.1
	9	Z.n.	3.2 (±0.2)	8.6 (±0.7)	0.6 (±0.5)	8.6 (±0.7)	7.3 (±1.6)	-	5.7 (±0.6)	18.0 (±1.2)	48.0 (±1.0)	3.9
Ammonium oxalate	10	A.a.	4.8 (±0.1)	2.3 (±1.0)	1.3 (±0.4)	7.2 (±3.9)	24.0 (±1.5)	13.6 (±3.9)	2.1 (±0.3)	32.5 (±2.7)	12.2 (±2.1)	19.8
	11	C.n	14.9 (±0.2)	2.6 (±1.0)	0.6 (±0.1)	18.9 (±1.5)	26.8 (±0.4)	7.1 (±1.7)	0.9 (±0.2)	24.4 (±1.0)	3.8 (±0.3)	41.6
	12	E.a.	6.2 (±0.5)	1.9 (±0.2)	-	58.2 (±1.6)	6.8 (±0.6)	-	1.9 (±0.2)	21.8 (±1.3)	3.2 (±1.0)	39.9
	13	P.a.	7.5 (±0.3)	1.2 (±0.1)	-	14.8 (±0.4)	27.1 (±0.6)	0.6 (±0.6)	2.5 (±0.5)	22.3 (±0.6)	24.0 (±1.3)	44.7

	14	P.s.	12.8 (±0.4)	6.4 (±0.7)	0.1 (±0.2)	13.9 (±0.4)	24.6 (±0.4)	2.8 (±0.2)	4.9 (±0.1)	20.6 (±0.7)	13.9 (±1.6)	48.8
	15	R.	21.9 (±1.0)	5.9 (±0.3)	7.9 (±0.6)	12.9 (±0.9)	15.9 (±1.0)	3.0 (±0.1)	3.6 (±0.7)	22.6 (±2.4)	6.3 (±0.5)	48.1
	16	T.h.	6.4 (±0.9)	4.2 (±1.1)	4.0 (±0.6)	22.2 (±6.8)	25.5 (±3.4)	17.9 (±2.3)	1.0 (±0.2)	16.3 (±2.8)	2.5 (±0.6)	33.3
	17	Z.m.	27.5 (±6.1)	4.6 (±0.7)	1.2 (±0.3)	15.9 (±3.0)	16.2 (±1.6)	5.4 (±0.5)	2.3 (±0.4)	24.0 (±0.9)	2.9 (±0.7)	40.5
	18	Z.n.	15.4 (±3.3)	9.0 (±0.2)	4.3 (±0.6)	13.6 (±5.5)	16.8 (±1.9)	12.8 (±5.2)	3.8 (±0.5)	19.6 (±2.5)	4.7 (±1.3)	35.9
Hydrochloric acid	19	A.a.	3.0 (±0.2)	0.7 (±0.1)	-	2.8 (±0.1)	78.3 (±2.3)	1.8 (±0.5)	0.6 (±0.1)	8.9 (±0.5)	3.9 (±1.6)	37.4
	20	C.n.	16.3 (±1.8)	1.4 (±0.7)	-	15.2 (±1.4)	36.0 (±1.8)	3.1 (±2.0)	1.8 (±0.2)	20.9 (±0.5)	5.3 (±1.2)	39.1
	21	E.a.	4.7 (±0.3)	5.3 (±2.9)	-	28.6 (±1.0)	3.7 (±0.3)	47.5 (±3.3)	-	8.9 (±0.5)	1.3 (±1.3)	14.7
	22	P.a.	4.7 (±0.2)	0.8 (±0.1)	-	7.4 (±0.2)	49.6 (±0.8)	8.0 (±1.1)	0.7 (±0.1)	8.6 (±0.2)	20.2 (±0.4)	19.9
	23	P.s.	5.2 (±0.1)	3.1 (±0.7)	-	6.7 (±0.3)	63.5 (±1.0)	10.9 (±1.8)	1.0 (±0.2)	6.0 (±0.3)	3.6 (±0.9)	28.6
	24	R.	11.1 (±0.7)	3.1 (±0.1)	0.6 (±0.1)	9.0 (±2.8)	53.0 (±1.6)	6.1 (±0.9)	1.6 (±0.1)	10.8 (±1.0)	4.8 (±0.5)	38.4
	25	T.h.	7.8 (±0.1)	2.5 (±0.2)	-	6.6 (±0.2)	66.5 (±0.4)	2.3 (±0.3)	0.9 (±0.2)	11.9 (±0.3)	1.5 (±0.4)	31.4
	26	Z.m.	16.8 (±3.5)	1.8 (±0.8)	-	7.8 (±5.1)	50.6 (±0.6)	3.0 (±0.6)	0.8 (±0.7)	15.6 (±1.8)	3.6 (±0.5)	19.1
	27	Z.n.	15.3 (±0.8)	13.3 (±0.6)	0.8 (±0.2)	6.1 (±1.7)	28.2 (±1.8)	16.4 (±2.7)	1.8 (±0.3)	13.2 (±0.5)	4.9 (±0.7)	29.4
Sodium carbonate	28	A.a.	3.8 (±0.1)	1.7 (±0.1)	-	6.3 (±0.9)	49.0 (±2.4)	-	2.2 (±0.7)	17.9 (±2.3)	19.1 (±1.4)	3.8
	29	C.n.	9.3 (±0.4)	0.8 (±0.2)	-	11.3 (±0.5)	62.5 (±0.6)	-	0.8 (±0.2)	12.7 (±0.4)	2.6 (±0.4)	17.3
	30	E.a.	10.5 (±0.2)	2.4 (±0.4)	0.4 (±0.4)	51.3 (±0.7)	5.0 (±0.5)	2.8 (±0.5)	1.9 (±0.4)	20.8 (±0.4)	4.9 (±0.4)	12.2

	31	P.a.	11.4 (±1.4)	1.2 (±0.1)	-	16.5 (±1.5)	25.1 (±2.6)	-	2.3 (±0.8)	18.9 (±2.6)	24.6 (±8.0)	4.8
	32	P.s.	11.2 (±0.6)	3.5 (±0.2)	-	17.0 (±1.1)	32.1 (±1.8)	0.9 (±0.8)	3.9 (±0.7)	17.6 (±2.3)	13.8 (±0.9)	4.2
	33	R.	13.3 (±0.6)	5.8 (±0.3)	-	9.7 (±0.4)	20.4 (±1.3)	-	5.0 (±0.6)	15.2 (±0.3)	30.6 (±3.0)	11.3
	34	T.h.	5.5 (±0.4)	1.5 (±0.1)	-	6.7 (±0.1)	68.3 (±1.0)	0.3 (±0.2)	1.1 (±0.2)	14.3 (±0.7)	2.3 (±0.3)	11.2
	35	Z.m.	3.1 (±0.5)	-	-	3.4 (±1.8)	78.2 (±1.5)	-	2.8 (±0.4)	4.7 (±0.4)	7.8 (±1.2)	11.9
	36	Z.n.	13.3 (±0.6)	23.9 (±1.8)	2.6 (±1.4)	9.2 (±0.4)	20.4 (±0.9)	0.7 (±0.1)	5.4 (±0.9)	18.6 (±1.3)	5.9 (±0.9)	30.7
Potassium hydroxide	37	A.a.	0.4 (±0.1)	0.6 (±0.1)	-	1.8 (±0.2)	89.4 (±0.6)	-	0.5 (±0.2)	4.1 (±0.4)	3.2 (±0.3)	2.8
	38	C.n.	5.2 (±1.7)	0.5 (±0.1)	-	8.0 (±0.1)	71.8 (±2.6)	-	1.0 (±0.1)	8.8 (±0.5)	4.7 (±1.2)	10.2
	39	E.a.	11.5 (±0.7)	1.7 (±0.6)	-	11.9 (±0.6)	53.2 (±1.9)	2.6 (±1.1)	0.2 (±0.2)	10.0 (±0.3)	8.9 (±0.5)	11.7
	40	P.a.	0.4 (±0.1)	0.1 (±0.1)	-	1.8 (±0.3)	41.1 (±4.3)	-	0.2 (±0.1)	2.0 (±0.7)	54.4 (±3.2)	1.0
	41	P.s.	7.2 (±0.9)	1.0 (±0.1)	-	8.1 (±0.3)	53.6 (±1.7)	-	3.5 (±0.5)	6.6 (±0.5)	20.0 (±0.4)	7.3
	42	R.	7.0 (±1.3)	1.4 (±0.1)	-	5.9 (±1.7)	31.3 (±1.0)	-	2.0 (±0.4)	7.6 (±0.5)	44.8 (±3.6)	10.0
	43	T.h.	2.6 (±0.4)	0.7 (±0.1)	-	3.6 (±0.1)	81.6 (±1.3)	-	0.6 (±0.1)	5.1 (±0.6)	5.8 (±0.2)	10.6
	44	Z.m.	17.5 (±6.1)	1.4 (±0.1)	-	12.0 (±1.0)	32.9 (±2.5)	-	5.5 (±2.9)	25.4 (±1.4)	5.3 (±0.5)	3.0
	45	Z.n.	4.6 (±1.0)	2.2 (±0.5)	-	3.2 (±0.6)	24.4 (±2.0)	-	1.1 (±0.4)	6.5 (±1.2)	58.0 (±4.7)	10.5

Table S4:

Constituent monosaccharide composition of the crude plant organs. Values are given in % (mol/mol).

Plant	Organ	Rha	Fuc	Ara	Xyl	Api	Man	Gal	Glc
Amphibolis antarctica	Leaves	2.5	1.2	3.7	48.0	12.4	2.2	13.2	16.8
	Rhizome	1.2	0.6	2.0	73.3	1.0	1.2	5.5	15.2
	Roots	3.8	1.9	5.8	63.7	4.8	2.2	9.2	8.6
Enhalus acoroides	Leaves	7.5	4.0	8.1	23.8	30.6	2.6	11.1	12.3
	Rhizome	3.6	1.6	5.9	8.6	8.7	1.6	3.6	66.4
	Roots	10.0	6.5	11.5	22.0	28.8	2.3	9.0	9.0
Zostera marina	Leaves	10.8	2.5	6.6	32.5	17.1	2.7	13.0	14.8
	Rhizome	9.6	2.9	4.0	15.5	8.4	2.5	8.3	48.8
	Roots	17.5	6.9	4.7	27.3	11.4	2.2	14.7	15.3
Posidonia australis	Leaves	9.2	1.4	3.6	48.3	18.5	1.3	6.0	11.7
	Rhizome	1.3	0.3	2.7	25.2	0.9	0.9	3.1	65.6
	Roots	4.7	0.6	10.3	45.3	2.4	1.8	9.0	25.9
	Sheaths	3.1	1.3	3.2	63.9	13.0	0.9	4.5	10.1

Table S5:

Uronic acid content of the crude organs, determined colorimetrically. Values are given in % (w/w) and are derived from three technical replicates.

Plant	Organ	Uronic acids
Amphibolis antarctica	Leaves	3.2 ± 0.1
	Rhizome	3.2 ± 0.1
	Roots	3.4 ± 0.2
Enhalus acoroides	Leaves	4.7 ± 0.6
	Rhizome	3.6 ± 1.1
	Roots	4.4 ± 0.6
Zostera marina	Leaves	6.7 ± 0.3
	Rhizome	8.7 ± 0.3
	Roots	7.7 ± 0.6
Posidonia australis	Leaves	3.8 ± 0.3
	Rhizome	2.4 ± 0.1
	Roots	0.1 ± 0.1
	Sheaths	2.7 ± 0.2

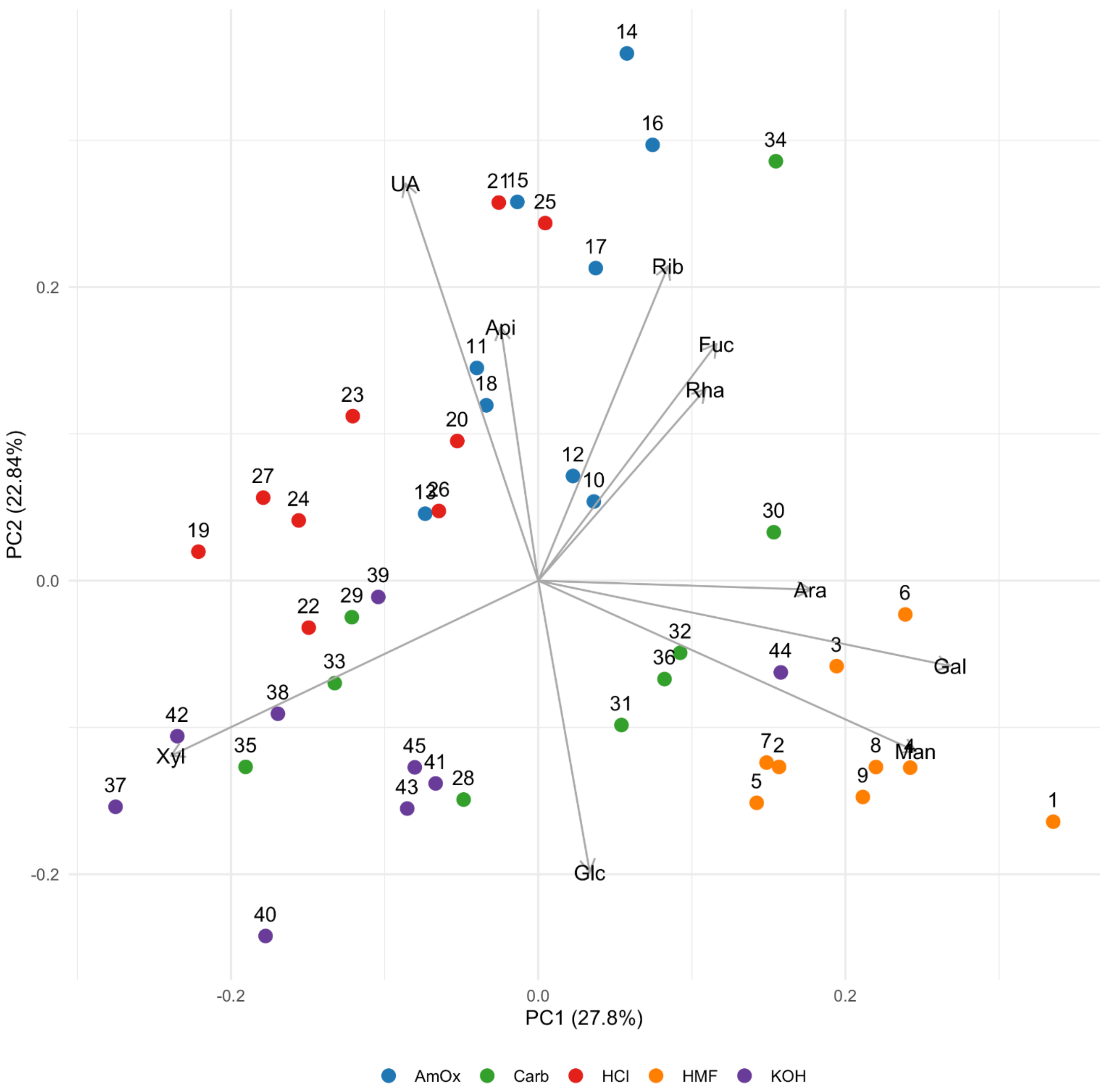

Fig. S1:

Principal Component Analysis (PCA) of the monosaccharide composition analysis for the fractionated extraction. The shown biplot illustrates the vectors of the loading variables, here the different monosaccharides with grey arrows. The sample numbers refer to Table S3.

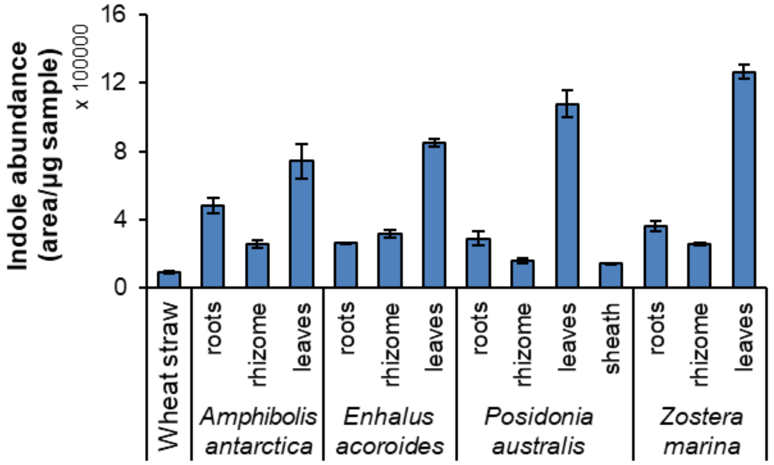

Fig. S2: Pyrolysis-GC-MS indole abundance, corrected for sample weight used for the analysis (µg). Average and standard deviation of technical triplicates. The wheat straw sample shown as reference is the same as previously published [1, 2].

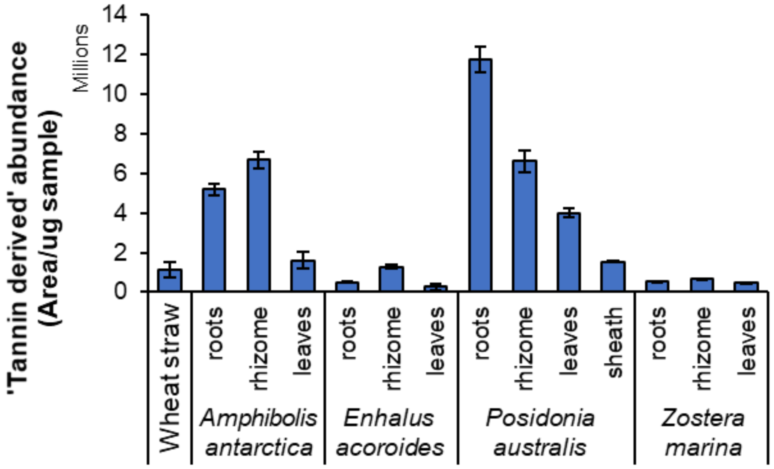

Fig. S3: Pyrolysis-GC-MS abundance of tannin-derived pyrolysis products, corrected for sample weight used for the analysis (µg). Average and standard deviation of technical triplicates. The wheat straw sample shown as reference is the same as previously published [1, 2].

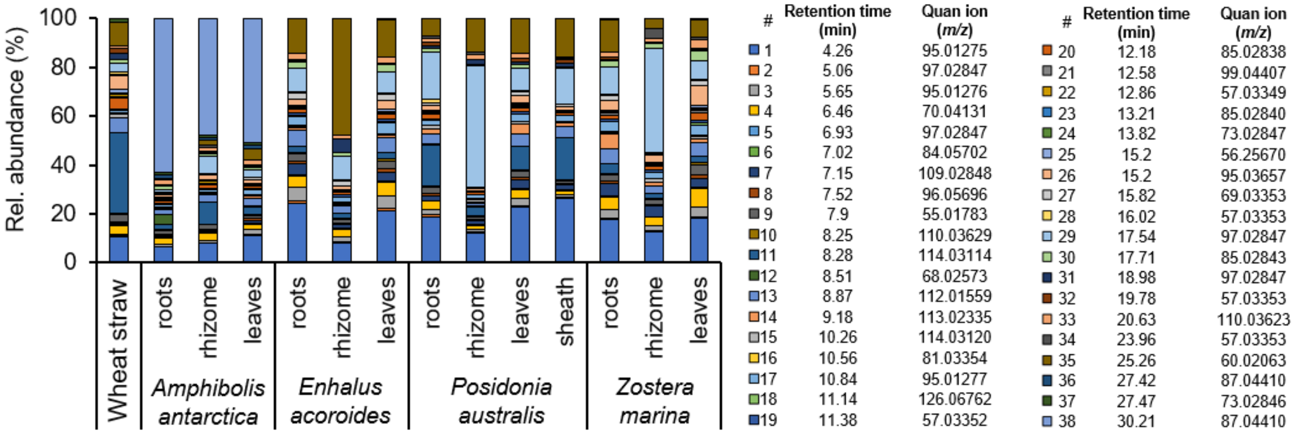

Fig. S4: Pyrolysis-GC-MS relative abundance of carbohydrate-derived pyrolysis products. Average of technical triplicates. The wheat straw sample shown as reference is the same as previously published [1, 2].

References:

1. van Erven G, de Visser R, Merkx DWH, Strolenberg W, de Gijsel P, Gruppen H, et al. Quantification of Lignin and Its Structural Features in Plant Biomass Using ^{13}C Lignin as Internal Standard for Pyrolysis-GC-SIM-MS. Anal Chem. 2017;89:10907–16.

2. van Erven G, de Visser R, de Waard P, van Berkel WJH, Kabel MA. Uniformly ^{13}C Labeled Lignin Internal Standards for Quantitative Pyrolysis−GC−MS Analysis of Grass and Wood. ACS Sustain Chem Eng. 2019;7:20070–6.
